# Comparison among anorexia nervosa adolescents with or without previous overweight, obese, and healthy adolescents

**DOI:** 10.3389/fpsyt.2024.1438829

**Published:** 2024-08-21

**Authors:** Federico Amianto, Francesca Sertori, Chiara Davico, Daniele Marcotulli, Benedetto Vitiello

**Affiliations:** ^1^ Neurosciences Department, University of Turin, Turin, Italy; ^2^ Department of Public Health and Pediatric Sciences, Section of Child and Adolescent Neuropsychiatry, University of Turin, Turin, Italy

**Keywords:** adolescence, anorexia nervosa, obesity, psychopathology, attachment

## Abstract

**Background:**

Anorexia nervosa (AN) and obesity (OB) are relevant concerns in adolescence. Despite their contrasting phenotypes, they share common pathogenic origins and may be present in the same individual at different times. We explored the psychopathology and attachment features of adolescents with AN who did (AN-ow) or did not (AN-nw) have previous overweight, compared with OB adolescents and healthy control (HC) adolescents.

**Method:**

In total, 148 female adolescents referred to an outpatient clinic for an eating disorder (66 for AN and 42 for OB) and 40 HCs were assessed using self-administered instruments that measured parenting, attachment, eating, and general psychopathology. Group differences were tested by analysis of covariance, and correlations between variables were examined.

**Results:**

AN-ow, AN-nw, and OB adolescents had greater interpersonal distrust and avoidance of relationships, compared with HC adolescents. AN-nw and AN-ow adolescents displayed a higher need for approval and a drive to thinness and interpersonal distrust, compared with HC adolescents. AN-ow adolescents displayed lower paternal care and higher ineffectiveness, asceticism, social insecurity, and depression, compared with HC adolescents. Compared with AN-nw adolescents, AN-ow adolescents felt more ineffective and more ascetic. The dynamics linking the psychopathological features clearly distinguished the four groups.

**Conclusions:**

AN-ow is a well-identified subtype of AN, with specific features that differ from AN-nw; some of these features are shared with OB. A therapeutic approach tailored to AN-ow adolescents should consider these features.

## Highlights

- Although AN and OB display opposite weight and feeding problems, they have common attachment and relational problems.
- AN-ow is characterized by higher ineffectiveness and asceticism compared with AN-nw, suggesting distinct developmental dynamics in these two AN subtypes.
- Characteristics that differ between AN subgroups and OB adolescents may be specific targets for psychotherapeutic interventions in AN. They may also be useful in prevention efforts and therapeutic interventions for childhood OB.

## 1 Introduction

Anorexia nervosa (AN) is a major health concern in female adolescents, and its incidence has risen during the coronavirus disease 2019 (COVID-19) pandemic (1, 2). Current treatments for AN are not consistently successful; the disorder often has severe clinical and social implications because of its protracted course, with high rates of chronicity, mortality, and relapse (3).

A large number of adolescents experience overweight or obesity (OB) before the onset of AN (4, 5). Up to one-third of adolescents with AN have a history of overweight or OB (6, 7). The increasing prevalence of overweight/OB has prompted multiple initiatives to mitigate the “epidemic of OB” (5). Weight loss is medically indicated to optimize health in patients with OB, but there is concern that weight reduction interventions may trigger the emergence of anorectic or bulimic behaviors (8). A few studies have compared AN with and without a history of overweight/OB to highlight the characteristics of each affected population, and the evidence is partially conflicting. Lebow et al. (6) reported that 36.7% of individuals with a restrictive eating disorder (ED) were obese before ED onset. They also had greater weight loss and a longer duration of illness before presentation; thus, they may have a poorer prognosis. Matthews et al. (4) confirmed that the AN population displayed greater ED severity and more anxiety or depression comorbidities, which were related to a more frequent history of weight-based teasing by peers; their results suggested specific preventive and therapeutic interventions in this subpopulation. Kennedy et al. (7) showed that although the degree of weight loss was greater in the AN subgroup with previous overweight/OB, the body mass index (BMI) at presentation was generally higher and the duration of treatment was not significantly different. To our knowledge, no study has explored the personality traits, eating psychopathology, or attachment dynamics that represent specific pathogenetic elements of these subtypes of AN. A comparison of the common features linking AN and OB may help define the problem.

OB is a complex condition that develops under the influence of biological and psychological factors; it often has lifelong implications for the health and psychological well-being of affected individuals (9–11). The prevalence of OB has been increasing worldwide (12)—in some countries, it constitutes a “social epidemic” (13). The prevalence of AN has dramatically increased in the Western world since the 1980s (14). Many studies have investigated the pathogenesis and features of OB individuals (15).

Although not classified by the Diagnostic and Statistical Manual of Mental Disorders (DSM)-5, OB is a clinical condition with frequent psychopathological comorbidities (16–18). High levels of eating psychopathology (e.g., drive to thinness, body dissatisfaction, bulimia, and maturity fears) are shared by individuals with OB or AN (18–20). High levels of ineffectiveness and dysfunctional relationship patterns are often found in patients with AN and OB (21–23).

Psychiatric comorbidities are frequent in OB (16, 18). High levels of deflected mood (16), obsessive-compulsive disorder (24), and binge eating disorder (25) have been reported in adolescents with OB. Similarly, adolescents with AN often display comorbid depression (21, 26) and comorbid obsessive-compulsive disorder (27, 28).

Disordered attachment was theorized as common root for the pathogenesis of the EDs (29). Attachment styles are associated with excessive control over food intake leading to underweight in patients with AN (28–31); they are associated with a lack of control leading to increased food intake in individuals with OB (32). Early parental experiences, including abandonment anxiety and unsafe attachment, are important pathogenic aspects in OB and AN (18, 19, 21, 33). Moreover, childhood abuse increases the risk of OB and AN (34–36).

The childhood attachment deficits may be responsible for a lack in emotional regulation in adolescence and adulthood (37). There is speculation that OB is associated with an inability to describe affective states, decipher emotions, and elaborate on mental representations (38). Therefore, OB individuals express their emotional needs by somatizing them as “concretized metaphors,” similar to AN individuals (39). This finding suggests that impaired emotional functioning, in particular a deficit in emotional identification and abnormal interoception, is a common origin of AN and OB (24).

Although it is difficult to compare the dietary behavior profile of AN (40) with the dietary behavior profile of OB, similar dysfunctional dietary attitudes are found in both conditions. Individuals with either disorder display false dietary beliefs, “all or nothing” thoughts, and the use of food to deal with negative emotions, leading to feeding practices that are either uncontrolled in OB or overcontrolled in AN, consistent with the typical attachment style of the disorder (24, 41). These dysfunctional eating habits are associated with anxiety, anger, fear, guilt, and a feeling of failure (24); they are dominated by uncontrolled emotionality leading to inadequate feeding control (15, 42).

There is evidence of common multifactorial pathogenesis in OB and AN, including eating and general psychopathology, as well as impaired relational and emotional functioning (43). Nevertheless, we hypothesized that some personality or psychopathological traits (e.g., persistence, bulimia, perfectionism, and insecure attachment), which distinguish AN individuals from OB individuals, may manifest in a distinct manner in AN with previous OB and AN without previous OB.

Therefore, the primary aim of this study was to explore attachment and psychopathological features among two subsets of adolescents with AN with or without a history of overweight/OB, compared with adolescents displaying OB and healthy controls (HCs). The goal was to understand which features are associated with the development of AN in overweight adolescents. This study also explored the dynamics connecting these features within each population to determine features specific to each condition. Findings from this study will help to distinguish possible AN subtypes based on their history of overweight and provide novel insights regarding tailored prevention and treatment strategies.

## 2 Methods

This study included 189 consecutive female patients aged 12–18 years who were admitted to the Outpatient Service for Adolescent Psychopathology and Eating Disorders of Regina Margherita Children’s Hospital in Turin, Italy, between January 1, 2019, and May 30, 2021. During the first visit, patients were assessed by a neuropsychiatrist using the Kiddie Schedule for Affective Disorders and Schizophrenia (K-SADS) for DSM-5 disorders (44); the diagnosis was confirmed by a second assessment 2 weeks later (second visit) and a discussion with the patient’s parents. Anthropometric measures were acquired by the clinician during the first visit.

Participants met the following inclusion criteria: female sex; age 12–18 years; completion of the administered psychometric tests; and diagnosis of AN, atypical AN, or primary OB (e.g., not secondary to another medical condition). The exclusion criteria were intellectual disability or a pervasive developmental disorder; neurodevelopmental disorder; and inability to comprehend and complete psychometric tests because of language or other cultural reasons. For the OB group the fulfillment of DSM 5 diagnostic criteria for an ED (e.g. BED) was also considered an exclusion criterion. The diagnosis of AN or atypical AN was based on current DSM-5 criteria (45), whereas the diagnosis of overweight or OB was based on body mass index percentile (BMI-P). After recruitment, the participants were classified into three groups: AN or atypical AN with previous normal weight (i.e., AN-nw), AN or atypical AN with a history of overweight (85^th^ < BMI-P < 94^th^; i.e., AN-ow), and OB (BMI-P ≥ 95^th^). The cutoff applied to distinguish AN and Atypical AN was the 10^th^ of the BMI-P distribution because it corresponds to the 18.5 BMI score described as threshold for AN diagnosis by the DSM 5.

The final clinical group consisted of 150 female adolescents: 44 with AN-nw, 24 with AN-ow, and 42 with OB. Nine adolescents had atypical AN in the AN-nw group, and 10 adolescents had atypical AN in the AN-ow group.

The HC group consisted of 40 female adolescents voluntarily recruited from middle and high schools in Turin, Italy. The participants were matched to the clinical group in terms of age, ethnicity, and sociocultural background; they were screened with the K-SADS to confirm the absence of any mental disorders. The HC group was included to represent the contemporary adolescent population without AN or OB to overcome possible limitations concerning normative populations.

### 2.1 Ethics

Both parents of all participants provided informed consent for their children to participate in the study. Refusal to participate did not affect treatment in any way. This study was conducted in accordance with the principles of the Declaration of Helsinki, as revised in October 2013.

### 2.2 Measures

All participants completed the following battery of self-evaluation measures, which were chosen based on previous evidence regarding the common origins of AN and BN (43):

1. Parental Bonding Instrument (PBI; 46): a measure of parental behavior as perceived by the child. This instrument consists of 25 items: 12 *care* items and 13 *protection* items. For mothers, the cutoff between high and low care scores is 27.0; it is 13.5 for protection. For fathers, the cutoffs are 24.0 for care and 12.5 for protection. Cronbach’s alpha for our sample = 0.71.
2. Attachment Style Questionnaire (ASQ; 47): a 40-item self-report questionnaire that assesses current attachment style and discriminates between secure and insecure attachment. The ASQ consists of five Likert scales derived from principal component analysis: *Confidence* (in self and others), *Discomfort with Closeness, Need for Approval* (from others)*, Preoccupation with Relationships*, and *Relationships as Secondary*. Cronbach’s alpha for our sample = 0.671.
3. Eating Disorder Inventory-2 (EDI-2; 48): a self-report inventory that measures disordered eating attitudes, behaviors, and personality traits common to individuals diagnosed with an ED. This test aims to assess the main psychopathological features related to EDs: *Drive to Thinness, Body Dissatisfaction, Bulimia, Inadequacy, Perfectionism, Interpersonal Distrust, Interoceptive Awareness, Asceticism, Impulsiveness, and Social Insecurity.* Cronbach’s alpha for our sample = 0.
4. Beck Depression Inventory-II (BDI-II; 49): a 21-item self-report questionnaire that measures the presence and severity of depressive symptoms. The test asks participants to place a mark next to the statement that best describes how they have felt over the past week for each item. Scores are summed across all items; cognitive depression, somatic depression, and total depression scores are computed. Cronbach’s alpha for our sample = 0.880.
5. Toronto Alexithymia Scale (TAS-20; 50): a 20-item self-assessment questionnaire used to assess alexithymia. The possible range of TAS-20 total scores is 20–100; participants are categorized as non-alexithymic (< 51), borderline (51–60), or alexithymic (≥ 61). Cronbach’s alpha for our sample = 0.790.

### 2.3 Procedure

During the first assessment visit at intake, a neuropsychiatrist administered the K-SADS semi-structured interview to the patient. During the interview, the clinician focused on weight problems, eating habits, and psychological distress. The patient’s age, length of education, BMI, maximum weight, age at ED onset, and age at onset of current symptoms were recorded. At the end of the visit, the patient was given self-administered psychometric tests to complete independently at home and return 1 week before the second visit. The completed tests were reviewed by the clinician, who returned the results to the patient during the second assessment visit. At the end of the visit, a general conclusive evaluation was provided using the Clinical Global Index, with particular attention to the possible diagnosis of an ED.

### 2.4 Statistical analysis

The chi-square test was used to test for differences between typical and atypical AN distributions in the AN-nw and AN-ow subgroups. Analysis of variance (ANOVA) was used to compare clinical and demographic measures (age, BMI, maximum weight, age at AN onset, and age at onset of concurrent symptoms) among the groups. Kruskal-Wallis H nonparametric test was used to perform BMI-P comparison among the groups. Analysis of covariance (ANCOVA) was used to compare attachment, eating psychopathology, general psychopathology, and alexithymia among the clinical groups (AN-nw, AN-ow, and OB) and the HC group; demographic variables that distinguished the four groups were used as confounding variables (age and BMI). *Post hoc* analysis was performed with the Bonferroni test.

Clinical (e.g., BMI, BMI-P, maximum weight, age at AN onset, and age at onset of concurrent symptoms), demographic (length of education), and psychometric (results of self-administered tests) variables in each clinical group were examined by Pearson correlation analysis to highlight possible groupwise differences in dynamics linking clinical conditions, attachment, and psychopathology. BMI-P and BMI were included in the analysis because BMI-P is used for ED classification, whereas BMI is more closely related to a patient’s self-perception.

Bonferroni correction based on the overall number of test variables considered was adopted with p <.007 for clinical and demographic variables (n = 7) and p <.002 for psychometric variables (n = 27); it was used for ANCOVA and Pearson correlation analysis to reduce type I error caused by the large number of variables. P <.05 was used for *post hoc* analyses.

Statistical analyses were performed using Statistical Package for Social Sciences software (SPSS 26.0, 2019).

## 3 Results

The difference between typical and atypical AN distributions in the AN-nw and AN-ow subgroups was not significant (**Table 1**).

**Table 1:** Clinical and sociodemographic features.

	(a)AN-nw(n= 44)mn±sd	(b)AN-ow(n= 24)mn±sd	(c)OB (n= 42)mn±sd	(d)HC(n= 40)mn±sd	F	P	η^2^	*Post-hoc*
Age	15.42±1.16	15.88±0.90	15.52±2.15	16.18±1.37	1.928	.000	0.013	–
BMI	16.28±1.96	17.81±1.67	34.95±7.07	21.11±2.71	162.032	.000	0.773	c>d>a,b
Years of education	9.49±1.51	9.83±0.86	8.62±1.81	10.93±1.54	15.430	.000	0.246	d>a,b,c
Maximum weight reached	50.62±7.64	71.27±9.02	103.41±31.57	–	68.525	.000	0.623	c>b>a
Age of onset of ED	13.58±2.43	13.77±2.65	9.41±4.06	–	17.539	.000	0.300	c<a,b
Age of onset of current symptoms	14.06±1.78	15.07±1.08	13.31±1.94	–	4.067	.023	0.148	–
					K-W test	P	fd	
BMI-P	10.03±13.01	19.65±18.27	98.21±2.10	45.77±5.23	131.027	.001	3	c>d>b>a
					χ^2^	P	fd	
Atypical AN	9 (20.4%)	10 (41.7%)	–	–	3.470	0.062	1	

AN-nw, anorexia with previous normal weight; AN-ow, anorexia with previous overweight/obesity; OB, obesity; HC, healthy controls; BMI, body mass index; K-W test, Kruskal-Wallis test; accepted significance p<.001.

### 3.1 Post hoc assessment of ANOVA used to compare clinical and sociodemographic features

**Table 1** shows the ANOVA comparison of clinical variables among the four groups. Here are reported only the results of the *post-hoc* analysis, the significance level is p<.001 for all variables. BMI was lower in the AN-nw and AN-ow than in the OB and controls, and higher in the OB than in the controls.

BMI-P was lower in the AN-nw and AN-ow than in the OB and controls, and higher in the OB than in the controls.

The AN-nw and OB had less education than the controls. Maximum weight reached was higher in the OB than in the AN-ow and AN-nw, and higher in the AN-ow than in the AN-nw. Disorder onset was earlier in the OB than AN-nw and AN-ow.

### 3.2 Post hoc assessment of ANCOVA used to compare AN-nw, AN-ow, OB, and HC groups

#### 3.2.1 Psychometric features

**Table 2** shows differences among the four groups in attachment, eating, general psychopathology, and degree of alexithymia.

**Table 2:** ANCOVA among personality and psychopathology features with age and BMI as confounding variables.

	(a)AN-nw(n= 44)mn±sd	(b)AN-ow(n= 24)mn±sd	(c)OB (n= 42)mn±sd	(d)HC (n= 40)mn±sd	F	P	η^2^	*Post-hoc*
*PBI*								
Paternal care	23.59±10.21	15.88±12.12	18.04±12.69	26.75±7.69	4.584	.004	0.107	b,c<d
*ASQ*								
Need for approval	25.83±6.43	28.53±5.513	21.29±7.96	20.73±5.87	11.687	.001	0.250	a,b>d; b>c
Relationships as secondary	20.38±5.21	21.29±6.38	20.19±7.40	15.83±4.71	5.724	.001	0.142	a,b,c>d
*EDI-2*								
Drive to thinness	10.17±8.21	15.29±4.97	8.90±5.76	5.73±6.00	9.648	.001	0.211	a,b>d; b>c
Bulimia	1.50±1.09	2.12±2.36	8.40±7.51	2.03±2.87	14.384	.001	0.285	c>a,b,d
Body dissatisfaction	11.0±8.10	14.12±7.63	19.53±6.27	10.0±7.67	8.626	.001	0.193	c>a,d
Ineffectiveness	7.60±6.41	15.76±9.31	10.03±6.53	4.70±5.76	10.746	.001	0.230	b,c>d; b>a
Interpersonal distrust	5.63±4.62	8.41±6.52	7.70±5.07	2.54±2.72	8.200	.001	0.186	a,b,c>d
Interoceptive awareness	11.47±7.74	14.94±7.66	9.07±5.83	5.30±5.40	8.993	.001	0.200	a,b>d; b>c
Asceticism	6.77±4.62	10.47±5.14	7.17±4.67	4.23±3.09	8.460	.001	0.190	b,c>d; a<b
Social insecurity	9.65±5.83	9.65±5.83	8.29±5.57	6.73±5.39	6.593	.001	0.156	b,c>d
*BDI*								
Total depression	17.67±13.17	27.53±15.27	19.35±11.72	10.75±9.22	8.156	.001	0.195	b,c>d; a<b
Cognitive depression	10.27±7.01	15.88±8.21	11.26±6.85	6.13±5.45	9.224	.001	0.211	b,c>d; a<b
Somatic depression	11.65±7.68	13.17±15.53	8.25±9.34	7.40±6.579	4.809	.002	0.125	b,c>d
*TAS*								
Difficulty identifying feelings	21.77±7.14	21.00±9.27	15.61±7.70	15.95±6.00	6.991	.001	0.166	a>c,d

AN-nw, anorexia with previous normal weight; AN-ow, anorexia with previous overweight/obesity; OB, obesity; HC, healthy controls; accepted significance p<.001.

Clinical groups had a greater tendency to view relationships as secondary compared with the HC (p <.011 for AN-nw; p <.009 for AN-ow; p <.020 for OB). The need for approval was greater in AN-nw (p <.011) and AN-ow (p <.001) than in HC, and higher in the AN-ow than in the OB (p <.003). Paternal care scores were lower in the AN-ow (p <.003) and OB (p <.006) than in HC.

Drive to thinness was higher in the AN-nw (p <.032) and AN-ow (p <.001) than in HC, and higher in the AN-ow than in the OB (p <.009). Bulimia was higher in the OB (p <.000) than in all other groups. Body dissatisfaction was higher in the OB (p <.000) than in the AN-nw and HC (p <.001).

The AN-ow (p <.001) and OB (p <.008) displayed greater ineffectiveness than HC. AN-ow also displayed greater ineffectiveness than AN-nw (p <.001). Interoceptive awareness was greater in the AN-nw and AN-ow than in HC (p <.001), and in the AN-ow than in the OB (p <.022). Asceticism was higher in the AN-ow (p <.001) and OB (p <.030) than in HC, and higher in the AN-ow (p <.001) than in the AN-nw. Social insecurity was greater in the AN-ow (p <.000) and OB (p <.002) than in HC. Interpersonal distrust was greater in the AN-nw (p <.033), AN-ow (p <.001), and OB (p <.001) than in HC.

The AN-ow and OB groups had more total depression (p <.001; p <.042), cognitive depression (p <.001; p <.024), and somatic depression (p <.041; p <.002) than HC. The AN-ow showed higher total depression (p <.046) and cognitive depression (p <.039) than the AN-nw. Difficulty identifying feelings was greater in the AN-nw than in the OB (p <.010) and HC (p <.007).

### 3.3 Pearson correlations among clinical, demographic, and psychometric features

The pattern of significant correlations (with p<.001) which have been found included 3 correlations for the AN-ow, none shared with the other groups; 4 for the OB, among which one shared with AN-nw and one with HC; 24 for the AN-nw and 25 for HC, among which 13 shared between the last two groups (between groups chi-square = 39.063; df = 3; p<0.001).

#### 3.3.1 Significant correlations in AN-ow and OB groups

**Table 3** displays the significant correlations among features in the AN-ow and the OB groups. Among AN-ow only three correlations were found with p <.001: maximum weight reached negatively correlated with difficulty identifying feelings, age at onset positively correlated with age at onset of current symptoms, and need for approval with body dissatisfaction.

**Table 3:** Pearson correlation in AN-ow and OB groups.

AN-ow		*r*	*p*
Age of onset of ED	Age of onset of current symptoms	.725	.000
*ASQ*			
Need for approval	(EDI) Body dissatisfaction	.747	.001
TAS			
Difficulty identifying feeling	Maximum weight reached	-.765	.001
OB			
*BMI-P*	CGI	.603	.001
*BMI*	Maximum weight reached	.897	.000
*EDI*			
Bulimia	(BDI) Somatic depression	.672	.000
	(BDI) Total depression	.637	.001
*BDI*			
Total depression	(EDI) Ineffectiveness	.637	.001

AN-ow, anorexia with previous overweight/obesity; OB, obesity; CGI, Clinical Global Impression; ASQ, Attachment Style Questionnaire; EDI, Eating Disorder Inventory-2; TAS, Toronto Alexithymia Scale-20; BDI, Beck Depression Inventory – II; BMI-P, body mass index percentile; BMI, body mass index; accepted significance p<.001.

Among OB three correlations were found with p <.001: BMI positively correlated with maximum weight, bulimia with somatic depression and total depression, total depression positively correlated with ineffectiveness.

#### 3.3.2 Significant correlations in the AN-nw group

**Table 4** displays the significant correlations among features in the AN-nw.

**Table 4:** Pearson correlation in AN-nw group.

AN-nw		r	P
Years of education	Age of onset ED	.523	.001
	Age of onset current symptoms	.653	.001
*BMI-P*	CGI (TAS) Total score	-.738 -.577	.001 .001
*ASQ*			
Need for approval	(EDI) Ineffectiveness	.645	.000
	(EDI) Social insecurity	.668	.000
	(BDI) Total depression	.603	.001
	(BDI) Cognitive depression	.645	.000
*EDI*			
Ineffectiveness	(BDI) Total depression	.695	.000
	(BDI) Cognitive depression	.714	.000
	(BDI) Somatic depression	.630	.000
Interoceptive awareness	(BDI) Total depression	.657	.000
	(BDI) Cognitive depression	.645	.000
	(BDI) Somatic depression	.628	.000
	(TAS) Difficulty identifying feeling	.781	.000
Asceticism	(TAS) Difficulty identifying feeling	.554	.001
Social insecurity	(BDI) Total depression	.740	.000
	(BDI) Cognitive depression	.739	.000
	(BDI) Somatic depression	.694	.000
	(TAS) Difficulty identifying feeling	.622	.000
*BDI*			
Total depression	(TAS) Difficulty identifying feeling	.707	.000
Cognitive depression	(TAS) Difficulty identifying feeling	.709	.000
	(TAS) Difficulty describing feelings	.590	.001
Somatic depression	(TAS) Difficulty identifying feeling	.659	.000

AN-nw, anorexia with previous normal weight; ASQ, Attachment Style Questionnaire; EDI, Eating Disorder Inventory-2; TAS, Toronto Alexithymia Scale-20; BDI, Beck Depression Inventory – II; BMI-P, body mass index percentile; accepted significance p<.001.

Among AN-nw twenty-four correlations were found with p <.001: they concerned years of education, BMI-P, need for approval, ineffectiveness, interoceptive awareness, asceticism, social insecurity, total, cognitive and somatic depression (see **Table 4** for details).

#### 3.3.3 Significant correlations in the HC group

**Table 5** displays the significant correlations among features in the HC.

**Table 5:** Pearson correlation in healthy controls (HC).

HC		r	P
*BMI*	(EDI) Drive to thinness	.676	.000
	(EDI) Body dissatisfaction	.601	.000
*PBI*			
Paternal care	(EDI) Social insecurity	-.577	.000
	(BDI) Somatic depression	-.514	.001
*EDI*			
Bulimia	(BDI) Total depression	.572	.000
	(BDI) Cognitive depression	.564	.000
	(BDI) Somatic depression	.521	.001
	(TAS) Difficulty identifying feeling	.540	.000
Body dissatisfaction	(BDI) Total depression	.521	.001
	(BDI) Cognitive depression	.531	.000
Ineffectiveness	(BDI) Total depression	.604	.000
	(BDI) Cognitive depression	.627	.000
	(BDI) Somatic depression	.511	.001
	(TAS) Difficulty identifying feeling	.568	.000
Interoceptive awareness	(BDI) Total depression	.575	.000
	(BDI) Cognitive depression	.562	.000
	(BDI) Somatic depression	.530	.000
	(TAS) Difficulty identifying feeling	.535	.000
Asceticism	(BDI) Cognitive depression	.469	.001
Social insecurity	(BDI) Total depression	.632	.000
	(BDI) Cognitive depression	.656	.000
	(BDI) Somatic depression	.534	.000
*BDI*			
Total depression	(TAS) Difficulty identifying feeling	.552	.000
Cognitive depression	(TAS) Difficulty identifying feeling	.552	.000
Somatic depression	(TAS) Difficulty identifying feeling	.490	.001

PBI, Parental Bonding Instrument; EDI, Eating Disorder Inventory-2; TAS, Toronto Alexithymia Scale-20; BDI, Beck Depression Inventory – II; BMI, body mass index; accepted significance p<.001.

Among HC twenty-five correlations were found with p <.001: they concerned BMI, paternal care, bulimia, body dissatisfaction, ineffectiveness, interoceptive awareness, asceticism, social insecurity, total, cognitive and somatic depression (see **Table 5** for details).

## 4 Discussion

This study explored differences among adolescents with a history of overweight/OB who later developed AN, adolescents with AN lacking previous overweight/OB, adolescents with OB, and HC adolescents. Despite the small sample size, many attachment and psychopathological features distinguished these adolescent subgroups.

All clinical groups (AN-nw, AN-ow, and OB) tended to view relationships as secondary and had higher interpersonal distrust, compared with HC adolescents. Viewing relationships as secondary is a dimension of attachment avoidance, which impairs the maintenance of stable and trusting relationships (47). Higher interpersonal distrust is a psychopathological trait commonly observed in EDs, which indicates difficulty trusting others (20). Both of these findings suggest that relational problems arising from distorted attachment attitudes are common in EDs and may share a common pathogenic role (29, 30). These typically afflict AN individuals during adolescence when emotional dysregulation is prevalent (51). In contrast, the OB group displayed an earlier age at onset of eating problems, which may precede or follow the development of relational problems in childhood.

Consistent with previous studies, the high need for approval, drive to thinness, and interoceptive awareness (a term used to define a difficulty in recognizing emotional states as opposed to the recognition of physical sensations) in our study were characteristic of adolescents with AN, relative to the OB and HC groups, regardless of previous weight status (18–20). Moreover, these features were significantly higher in AN-ow than in OB adolescents; thus, they were characteristic of overweight/OB children who developed AN in adolescence instead of maintaining their weight status. The finding that these features represent specific risk factors for AN is supported by many studies. The need for approval is linked to the development of AN in affected siblings, compared with unaffected siblings, in the same family (22). Additionally, the need for approval is independently related to body dissatisfaction in AN (19); it links AN psychopathology with obsessive-compulsive symptoms (28). The drive to thinness is a specific feature that distinguishes AN from other EDs; it represents a behavior closely related to weight loss in adolescents with AN (20). Interoceptive awareness is strictly related to the psychosomatic mechanisms of AN pathogenesis as a “concretized metaphor” of inner mental illness in these individuals (39).

Difficulty identifying feelings was the only specific feature in adolescents with AN-nw. Alexithymic traits are frequently associated with AN; this association has been supported by neuroimaging findings (52). High levels of alexithymia have been identified as a contributing factor to AN pathogenesis, in the form of a “concretized metaphor” (39). Even if these levels are not essential for ED onset (29), they may promote an extreme manifestation of this psychosomatic mechanism, leading to the development of the AN-nw subtype. The specificity of this feature for this subgroup, identified by childhood history rather than DSM-5 criteria, may explain the conflicting findings regarding the pathogenic role of alexithymia in AN (29). If future research confirms this finding, more specific psychotherapeutic approaches may be targeted toward the AN-nw subgroup.

AN-ow is the most characterized subgroup. Similar to OB adolescents, AN-ow adolescents displayed lower paternal care and greater interpersonal distrust compared with HC adolescents; they also displayed greater ineffectiveness and asceticism, compared with AN-nw and HC adolescents. In addition to the greater alexithymia in AN-nw adolescents, these are the characteristic features that most clearly distinguished our AN subgroups according to weight history; they support two well-differentiated psychopathological landscapes that may help tailor treatment approaches in these AN subgroups. The finding that both groups (AN-ow and OB) with childhood overweight/OB recalled low paternal care is suggestive. Recent evidence emphasizes that unfavorable parenting in childhood (i.e., affectionless control) represents a specific risk factor for the development of overweight/OB (18, 35). AN-ow adolescents may develop AN in relation to psychopathological features specific to this disorder, but this early risk factor may be unresolved. Therefore, because this feature characterizes these adolescents after the development of AN, it may require specific psychological clarification during treatment.

Higher interpersonal distrust manifests in a manner similar to low paternal care; thus, an association between these two risk factors cannot be excluded (18). This psychopathological feature reinforces the severity of relational dysfunction as a risk factor for the development of AN-ow, as demonstrated among all ED groups in our study (29, 30). Paternal attachment problems and social insecurity may contribute to weight gain in childhood because of inadequate stress hormone modulation in relational situations, thereby possibly exacerbating dietary problems (18, 53).

Although ineffectiveness is a well-known psychopathological trait in individuals with an ED, to our knowledge, no study has associated it with any specific ED (48). Ineffectiveness refers to the sense of being incapable of producing a desired result, a lack of determination, and the absence of self-purpose (48). Asceticism refers to the renunciation of pleasure and gratification to strengthen willpower and directionality (48); it is typically associated with AN (20). Recent research suggests that adolescents with binge-purge symptoms have higher levels of asceticism compared with adolescents who display restrictive symptoms (54). These traits may be linked to a psychopathological dynamic in which the sense of ineffectiveness is mimicked by ascetic traits that produce restrictive symptoms, but without the sense of effectiveness that typically characterizes AN individuals after weight loss. This outcome may cause greater depression in AN-ow adolescents than in AN-nw adolescents. Ineffectiveness and asceticism are linked to depression (16, 21, 26). Depression is an internalizing strategy to manage emotional disorders that are closely related to asceticism (55); our study showed that it was particularly prevalent among AN-ow and OB adolescents. Future longitudinal studies may provide further insight into this dynamic perspective.

The correlations between attachment and psychopathology support the existence of specific dynamics in AN groups. These correlations were very limited in the AN-ow group (possibly because of the small sample size) and focused on the relationship between the need for approval and body dissatisfaction, consistent with the findings of Abbate Daga et al. (19). Additionally, alexithymic traits and maximum weight were related, supporting the “concretized metaphor” mechanism in this subgroup (39).

The correlations in the AN-nw group were extensive and created a complex landscape in which attachment and psychopathology features were closely intertwined. As suggested by the results of a previous study (18), our findings support a more linear pathogenesis in AN-nw than AN-ow and indicate greater overlap between psychopathological processes conducive to OB and processes conducive to AN-ow.

## 5 Limitations

The limitations of the present study include its relatively small sample size, particularly in the AN-ow subgroup. This may have introduced selection biases and reduced the statistical power of the analyses; thus, replication in larger samples is required. Additionally, the relatively small number of participants did not allow differentiation between AN diagnostic subgroups (restriction/binge-purging) or between AN and atypical AN. A larger sample size would permit further elucidation of the relationship between childhood OB history and the AN diagnostic subtypes that develop during adolescence. The use of self-administered instruments may have produced reporting/recall bias. Future in-depth investigations should include semi-structured interviews and be supported by hetero-administered instruments (e.g., the Child Behavior Checklist administered to parents). Finally, the cross-sectional nature of the study did not permit exploration of the direction of the relationship between attachment and psychopathological features. Cohort studies that follow overweight and normal-weight girls until adolescence may be useful to elucidate the evolution of clinical, eating, and general psychopathological features prior to AN onset. Such studies could help to define causal hypotheses linking childhood weight history, attachment, and psychopathology to AN onset. Despite these limitations, this study is one of the first attempts to distinguish AN individuals based on disorder history rather than current symptoms, potentially providing therapeutic insights.

## 6 Conclusion

Although AN and OB display opposite weight and feeding problems, they share attachment and relational problems, which may be linked to common pathogenic pathways. AN-ow and AN-nw adolescents formed distinct subgroups among adolescents with AN. Both subgroups are characterized by psychopathological features specific to AN, such as a high need for approval, a drive to thinness, and interoceptive awareness. However, AN-ow adolescents were distinct from AN-nw adolescents in that they showed a greater sense of ineffectiveness linked to greater asceticism and social insecurity, as well as lower paternal care. These features were related to greater depression levels in AN-ow adolescents, a feature that is typical of adolescents with overweight. The differences between AN-ow and AN-nw adolescents may represent specific targets for psychotherapeutic interventions in AN, which should be carefully tailored to each individual’s pathogenic history (56). Psychopathological between-group differences among AN subgroups may explain some reports of greater distress in AN-ow adolescents (5). For example, AN-ow adolescents may require support in clarifying the dynamics that contributed to the development of childhood overweight (which also contribute to OB), such as low paternal care, high ineffectiveness, asceticism, social insecurity, and depressive feelings; these dynamics may be less relevant for AN-nw adolescents. Moreover, the differences identified here may be useful in prevention efforts and therapeutic interventions for childhood OB regarding the detection of specific attachment and eating psychopathology markers in childhood and early adolescence (57).

## Data Availability

The raw data supporting the conclusions of this article will be made available by the authors, without undue reservation.
