# Supplementary material for: Comparison among anorexia nervosa adolescents with or without previous overweight, obese, and healthy adolescents
Source: Front Psychiatry. 2024 Aug 21;15:1438829. doi: 10.3389/fpsyt.2024.1438829 (PMC11371598; doi:10.3389/fpsyt.2024.1438829)
Supplement: Supplementary file 1 [file Table1.docx]

| **Correlations AN-nw group** | | | | | | | | | |
| --- | --- | --- | --- | --- | --- | --- | --- | --- | --- |
|  | | age | education | bmi | BMI_P | Drive to Thinness | Bulimia | Body Dissatisfaction | Ineffectiveness |
| age | Pearson Correlation | 1 | ,990^**^ | ,106 | -,257 | ,063 | -,026 | ,044 | -,015 |
|  | Sign. two tailed |  | <,001 | ,492 | ,092 | ,739 | ,890 | ,819 | ,936 |
|  | N | 44 | 44 | 44 | 44 | 44 | 30 | 30 | 30 |
| education | Pearson Correlation | ,990^**^ | 1 | ,119 | -,268 | -,063 | -,007 | ,008 | -,099 |
|  | Sign. two tailed | <,001 |  | ,448 | ,082 | ,744 | ,969 | ,968 | ,609 |
|  | N | 44 | 44 | 44 | 44 | 44 | 44 | 44 | 44 |
| bmi | Pearson Correlation | ,106 | ,119 | 1 | ,778^**^ | -,036 | ,424^*^ | -,057 | -,005 |
|  | Sign. two tailed | ,492 | ,448 |  | <,001 | ,852 | ,020 | ,763 | ,980 |
|  | N | 44 | 44 | 44 | 44 | 44 | 44 | 44 | 44 |
| BMI_P | Pearson Correlation | -,257 | -,268 | ,778^**^ | 1 | -,030 | ,272 | ,009 | -,103 |
|  | Sign. two tailed | ,092 | ,082 | <,001 |  | ,876 | ,146 | ,964 | ,589 |
|  | N | 44 | 44 | 44 | 44 | 44 | 44 | 44 | 44 |
| Drive to Thinness | Pearson Correlation | ,063 | -,063 | -,036 | -,030 | 1 | ,031 | ,698^**^ | ,333 |
|  | Sign. two tailed | ,739 | ,744 | ,852 | ,876 |  | ,871 | <,001 | ,073 |
|  | N | 44 | 44 | 44 | 44 | 44 | 44 | 44 | 44 |
| Bulimia | Pearson Correlation | -,026 | -,007 | ,424^*^ | ,272 | ,031 | 1 | ,140 | ,308 |
|  | Sign. two tailed | ,890 | ,969 | ,020 | ,146 | ,871 |  | ,461 | ,098 |
|  | N | 44 | 44 | 44 | 44 | 44 | 44 | 44 | 44 |
| Body Dissatisfaction | Pearson Correlation | ,044 | ,008 | -,057 | ,009 | ,698^**^ | ,140 | 1 | ,438^*^ |
|  | Sign. two tailed | ,819 | ,968 | ,763 | ,964 | <,001 | ,461 |  | ,016 |
|  | N | 44 | 44 | 44 | 44 | 44 | 44 | 44 | 44 |
| Ineffectiveness | Pearson Correlation | -,015 | -,099 | -,005 | -,103 | ,333 | ,308 | ,438^*^ | 1 |
|  | Sign. two tailed | ,936 | ,609 | ,980 | ,589 | ,073 | ,098 | ,016 |  |
|  | N | 44 | 44 | 44 | 44 | 44 | 44 | 44 | 44 |
| Social Insecurity | Pearson Correlation | ,043 | ,000 | -,236 | -,289 | ,544^**^ | -,064 | ,586^**^ | ,599^**^ |
|  | Sign. two tailed | ,823 | ,999 | ,209 | ,121 | ,002 | ,737 | <,001 | <,001 |
|  | N | 44 | 44 | 44 | 44 | 44 | 44 | 44 | 44 |
| Asceticism | Pearson Correlation | -,177 | -,337 | -,227 | -,177 | ,572^**^ | ,254 | ,546^**^ | ,430^*^ |
|  | Sign. two tailed | ,351 | ,074 | ,228 | ,350 | <,001 | ,176 | ,002 | ,018 |
|  | N | 44 | 44 | 44 | 44 | 44 | 44 | 44 | 44 |
| Interosceptive Awareness | Pearson Correlation | -,023 | -,184 | -,187 | -,191 | ,539^**^ | ,089 | ,338 | ,697^**^ |
|  | Sign. two tailed | ,902 | ,340 | ,323 | ,312 | ,002 | ,639 | ,067 | <,001 |
|  | N | 44 | 44 | 44 | 44 | 44 | 44 | 44 | 44 |
| Interpersonal Distrust | Pearson Correlation | -,003 | -,077 | -,280 | -,324 | ,394^*^ | -,144 | ,347 | ,295 |
|  | Sign. two tailed | ,986 | ,690 | ,134 | ,080 | ,031 | ,448 | ,061 | ,114 |
|  | N | 44 | 44 | 44 | 44 | 44 | 44 | 44 | 44 |
| Difficulty in Identifying Feelings | Pearson Correlation | ,106 | -,074 | -,455^*^ | -,533^**^ | ,525^**^ | ,013 | ,273 | ,523^**^ |
|  | Sign. two tailed | ,578 | ,703 | ,011 | ,002 | ,003 | ,947 | ,144 | ,003 |
|  | N | 44 | 44 | 44 | 44 | 44 | 44 | 44 | 44 |
| PATERNAL CARE | Pearson Correlation | ,045 | ,104 | -,226 | -,149 | -,109 | -,194 | -,099 | -,101 |
|  | Sign. two tailed | ,817 | ,599 | ,239 | ,440 | ,573 | ,313 | ,611 | ,601 |
|  | N | 44 | 44 | 44 | 44 | 44 | 44 | 44 | 44 |
| Total Depression | Pearson Correlation | ,230 | ,112 | -,146 | -,251 | ,474^**^ | ,005 | ,387^*^ | ,695^**^ |
|  | Sign. two tailed | ,221 | ,564 | ,443 | ,182 | ,008 | ,979 | ,035 | <,001 |
|  | N | 44 | 44 | 44 | 44 | 44 | 44 | 44 | 44 |
| Cognitive Depression | Pearson Correlation | ,261 | ,117 | -,214 | -,309 | ,481^**^ | -,052 | ,414^*^ | ,714^**^ |
|  | Sign. two tailed | ,164 | ,545 | ,256 | ,097 | ,007 | ,787 | ,023 | <,001 |
|  | N | 44 | 44 | 44 | 44 | 44 | 44 | 44 | 44 |
| Somatic Depression | Pearson Correlation | ,183 | ,099 | -,063 | -,172 | ,437^*^ | ,065 | ,333 | ,630^**^ |
|  | Sign. two tailed | ,333 | ,610 | ,741 | ,363 | ,016 | ,733 | ,072 | <,001 |
|  | N | 44 | 44 | 44 | 44 | 44 | 44 | 44 | 44 |

| **Correlations** | | | | | | | | | |
| --- | --- | --- | --- | --- | --- | --- | --- | --- | --- |
|  | | Social Insecurity | Asceticism | Interosceptive Awareness | Interpersonal Distrust | Difficulty in Identifying Feelings | PATERNAL CARE | Total Depression | Cognitive Depression |
| age | Pearson Correlation | ,043 | -,177 | -,023 | -,003 | ,106 | ,045 | ,230 | ,261 |
|  | Sign. two tailed | ,823 | ,351 | ,902 | ,986 | ,578 | ,817 | ,221 | ,164 |
|  | N | 44 | 44 | 44 | 44 | 44 | 44 | 44 | 44 |
| education | Pearson Correlation | ,000 | -,337 | -,184 | -,077 | -,074 | ,104 | ,112 | ,117 |
|  | Sign. two tailed | ,999 | ,074 | ,340 | ,690 | ,703 | ,599 | ,564 | ,545 |
|  | N | 44 | 44 | 44 | 44 | 44 | 44 | 44 | 44 |
| bmi | Pearson Correlation | -,236 | -,227 | -,187 | -,280 | -,455^*^ | -,226 | -,146 | -,214 |
|  | Sign. two tailed | ,209 | ,228 | ,323 | ,134 | ,011 | ,239 | ,443 | ,256 |
|  | N | 44 | 44 | 44 | 44 | 44 | 44 | 44 | 44 |
| BMI_P | Pearson Correlation | -,289 | -,177 | -,191 | -,324 | -,533^**^ | -,149 | -,251 | -,309 |
|  | Sign. two tailed | ,121 | ,350 | ,312 | ,080 | ,002 | ,440 | ,182 | ,097 |
|  | N | 44 | 44 | 44 | 44 | 44 | 44 | 44 | 44 |
| Drive to Thinness | Pearson Correlation | ,544^**^ | ,572^**^ | ,539^**^ | ,394^*^ | ,525^**^ | -,109 | ,474^**^ | ,481^**^ |
|  | Sign. two tailed | ,002 | <,001 | ,002 | ,031 | ,003 | ,573 | ,008 | ,007 |
|  | N | 44 | 44 | 44 | 44 | 44 | 44 | 44 | 44 |
| Bulimia | Pearson Correlation | -,064 | ,254 | ,089 | -,144 | ,013 | -,194 | ,005 | -,052 |
|  | Sign. two tailed | ,737 | ,176 | ,639 | ,448 | ,947 | ,313 | ,979 | ,787 |
|  | N | 44 | 44 | 44 | 44 | 44 | 44 | 44 | 44 |
| Body Dissatisfaction | Pearson Correlation | ,586^**^ | ,546^**^ | ,338 | ,347 | ,273 | -,099 | ,387^*^ | ,414^*^ |
|  | Sign. two tailed | <,001 | ,002 | ,067 | ,061 | ,144 | ,611 | ,035 | ,023 |
|  | N | 44 | 44 | 44 | 44 | 44 | 44 | 44 | 44 |
| Ineffectiveness | Pearson Correlation | ,599^**^ | ,430^*^ | ,697^**^ | ,295 | ,523^**^ | -,101 | ,695^**^ | ,714^**^ |
|  | Sign. two tailed | <,001 | ,018 | <,001 | ,114 | ,003 | ,601 | <,001 | <,001 |
|  | N | 44 | 44 | 44 | 44 | 44 | 44 | 44 | 44 |
| Social Insecurity | Pearson Correlation | 1 | ,551^**^ | ,617^**^ | ,757^**^ | ,622^**^ | -,054 | ,740^**^ | ,739^**^ |
|  | Sign. two tailed |  | ,002 | <,001 | <,001 | <,001 | ,781 | <,001 | <,001 |
|  | N | 44 | 44 | 44 | 44 | 44 | 44 | 44 | 44 |
| Asceticism | Pearson Correlation | ,551^**^ | 1 | ,535^**^ | ,514^**^ | ,554^**^ | -,110 | ,418^*^ | ,442^*^ |
|  | Sign. two tailed | ,002 |  | ,002 | ,004 | ,001 | ,569 | ,021 | ,015 |
|  | N | 44 | 44 | 44 | 44 | 44 | 44 | 44 | 44 |
| Interosceptive Awareness | Pearson Correlation | ,617^**^ | ,535^**^ | 1 | ,380^*^ | ,781^**^ | -,123 | ,657^**^ | ,645^**^ |
|  | Sign. two tailed | <,001 | ,002 |  | ,038 | <,001 | ,524 | <,001 | <,001 |
|  | N | 44 | 44 | 44 | 44 | 44 | 44 | 44 | 44 |
| Interpersonal Distrust | Pearson Correlation | ,757^**^ | ,514^**^ | ,380^*^ | 1 | ,500^**^ | -,013 | ,530^**^ | ,500^**^ |
|  | Sign. two tailed | <,001 | ,004 | ,038 |  | ,005 | ,947 | ,003 | ,005 |
|  | N | 44 | 44 | 44 | 44 | 44 | 44 | 44 | 44 |
| Difficulty in Identifying Feelings | Pearson Correlation | ,622^**^ | ,554^**^ | ,781^**^ | ,500^**^ | 1 | ,046 | ,707^**^ | ,709^**^ |
|  | Sign. two tailed | <,001 | ,001 | <,001 | ,005 |  | ,814 | <,001 | <,001 |
|  | N | 44 | 44 | 44 | 44 | 44 | 44 | 44 | 44 |
| PATERNAL CARE | Pearson Correlation | -,054 | -,110 | -,123 | -,013 | ,046 | 1 | -,049 | -,012 |
|  | Sign. two tailed | ,781 | ,569 | ,524 | ,947 | ,814 |  | ,802 | ,950 |
|  | N | 44 | 44 | 44 | 44 | 44 | 44 | 44 | 44 |
| Total Depression | Pearson Correlation | ,740^**^ | ,418^*^ | ,657^**^ | ,530^**^ | ,707^**^ | -,049 | 1 | ,971^**^ |
|  | Sign. two tailed | <,001 | ,021 | <,001 | ,003 | <,001 | ,802 |  | <,001 |
|  | N | 44 | 44 | 44 | 44 | 44 | 44 | 44 | 44 |
| Cognitive Depression | Pearson Correlation | ,739^**^ | ,442^*^ | ,645^**^ | ,500^**^ | ,709^**^ | -,012 | ,971^**^ | 1 |
|  | Sign. two tailed | <,001 | ,015 | <,001 | ,005 | <,001 | ,950 | <,001 |  |
|  | N | 44 | 44 | 44 | 44 | 44 | 44 | 44 | 44 |
| Somatic Depression | Pearson Correlation | ,694^**^ | ,367^*^ | ,628^**^ | ,528^**^ | ,659^**^ | -,084 | ,967^**^ | ,878^**^ |
|  | Sign. two tailed | <,001 | ,046 | <,001 | ,003 | <,001 | ,663 | <,001 | <,001 |
|  | N | 44 | 44 | 44 | 44 | 44 | 44 | 44 | 44 |

| **Correlations** | | | | |  |  |  |  |  |  |  |  |  |  |  |  |  |  |
| --- | --- | --- | --- | --- | --- | --- | --- | --- | --- | --- | --- | --- | --- | --- | --- | --- | --- | --- |
|  | | Somatic Depression | | |  |  |  |  |  |  |  |  |  |  |  |  |  |  |
| age | Pearson Correlation | ,183 | | |  |  |  |  |  |  |  |  |  |  |  |  |  |  |
|  | Sign. two tailed | ,333 | | |  |  |  |  |  |  |  |  |  |  |  |  |  |  |
|  | N | 44 | | |  |  |  |  |  |  |  |  |  |  |  |  |  |  |
| education | Pearson Correlation | ,099 | | |  |  |  |  |  |  |  |  |  |  |  |  |  |  |
|  | Sign. two tailed | ,610 | | |  |  |  |  |  |  |  |  |  |  |  |  |  |  |
|  | N | 44 | | |  |  |  |  |  |  |  |  |  |  |  |  |  |  |
| bmi | Pearson Correlation | -,063 | | |  |  |  |  |  |  |  |  |  |  |  |  |  |  |
|  | Sign. two tailed | ,741 | | |  |  |  |  |  |  |  |  |  |  |  |  |  |  |
|  | N | 44 | | |  |  |  |  |  |  |  |  |  |  |  |  |  |  |
| BMI_P | Pearson Correlation | -,172 | | |  |  |  |  |  |  |  |  |  |  |  |  |  |  |
|  | Sign. two tailed | ,363 | | |  |  |  |  |  |  |  |  |  |  |  |  |  |  |
|  | N | 44 | | |  |  |  |  |  |  |  |  |  |  |  |  |  |  |
| Drive to Thinness | Pearson Correlation | ,437^*^ | | |  |  |  |  |  |  |  |  |  |  |  |  |  |  |
|  | Sign. two tailed | ,016 | | |  |  |  |  |  |  |  |  |  |  |  |  |  |  |
|  | N | 44 | | |  |  |  |  |  |  |  |  |  |  |  |  |  |  |
| Bulimia | Pearson Correlation | ,065 | | |  |  |  |  |  |  |  |  |  |  |  |  |  |  |
|  | Sign. two tailed | ,733 | | |  |  |  |  |  |  |  |  |  |  |  |  |  |  |
|  | N | 44 | | |  |  |  |  |  |  |  |  |  |  |  |  |  |  |
| Body Dissatisfaction | Pearson Correlation | ,333 | | |  |  |  |  |  |  |  |  |  |  |  |  |  |  |
|  | Sign. two tailed | ,072 | | |  |  |  |  |  |  |  |  |  |  |  |  |  |  |
|  | N | 44 | | |  |  |  |  |  |  |  |  |  |  |  |  |  |  |
| Ineffectiveness | Pearson Correlation | ,630^**^ | | |  |  |  |  |  |  |  |  |  |  |  |  |  |  |
|  | Sign. two tailed | <,001 | | |  |  |  |  |  |  |  |  |  |  |  |  |  |  |
|  | N | 44 | | |  |  |  |  |  |  |  |  |  |  |  |  |  |  |
| Social Insecurity | Pearson Correlation | ,694^**^ | | |  |  |  |  |  |  |  |  |  |  |  |  |  |  |
|  | Sign. two tailed | <,001 | | |  |  |  |  |  |  |  |  |  |  |  |  |  |  |
|  | N | 44 | | |  |  |  |  |  |  |  |  |  |  |  |  |  |  |
| Asceticism | Pearson Correlation | ,367^*^ | | |  |  |  |  |  |  |  |  |  |  |  |  |  |  |
|  | Sign. two tailed | ,046 | | |  |  |  |  |  |  |  |  |  |  |  |  |  |  |
|  | N | 44 | | |  |  |  |  |  |  |  |  |  |  |  |  |  |  |
| Interosceptive Awareness | Pearson Correlation | ,628^**^ | | |  |  |  |  |  |  |  |  |  |  |  |  |  |  |
|  | Sign. two tailed | <,001 | | |  |  |  |  |  |  |  |  |  |  |  |  |  |  |
|  | N | 44 | | |  |  |  |  |  |  |  |  |  |  |  |  |  |  |
| Interpersonal Distrust | Pearson Correlation | ,528^**^ | | |  |  |  |  |  |  |  |  |  |  |  |  |  |  |
|  | Sign. two tailed | ,003 | | |  |  |  |  |  |  |  |  |  |  |  |  |  |  |
|  | N | 44 | | |  |  |  |  |  |  |  |  |  |  |  |  |  |  |
| Difficulty in Identifying Feelings | Pearson Correlation | ,659^**^ | | |  |  |  |  |  |  |  |  |  |  |  |  |  |  |
|  | Sign. two tailed | <,001 | | |  |  |  |  |  |  |  |  |  |  |  |  |  |  |
|  | N | 44 | | |  |  |  |  |  |  |  |  |  |  |  |  |  |  |
| PATERNAL CARE | Pearson Correlation | -,084 | | |  |  |  |  |  |  |  |  |  |  |  |  |  |  |
|  | Sign. two tailed | ,663 | | |  |  |  |  |  |  |  |  |  |  |  |  |  |  |
|  | N | 44 | | |  |  |  |  |  |  |  |  |  |  |  |  |  |  |
| Total Depression | Pearson Correlation | ,967^**^ | | |  |  |  |  |  |  |  |  |  |  |  |  |  |  |
|  | Sign. two tailed | <,001 | | |  |  |  |  |  |  |  |  |  |  |  |  |  |  |
|  | N | 44 | | |  |  |  |  |  |  |  |  |  |  |  |  |  |  |
| Cognitive Depression | Pearson Correlation | ,878^**^ | | |  |  |  |  |  |  |  |  |  |  |  |  |  |  |
|  | Sign. two tailed | <,001 | | |  |  |  |  |  |  |  |  |  |  |  |  |  |  |
|  | N | 44 | | |  |  |  |  |  |  |  |  |  |  |  |  |  |  |
| Somatic Depression | Pearson Correlation | 1 | | |  |  |  |  |  |  |  |  |  |  |  |  |  |  |
|  | Sign. two tailed |  | | |  |  |  |  |  |  |  |  |  |  |  |  |  |  |
|  | N | 44 | | |  |  |  |  |  |  |  |  |  |  |  |  |  |  |
|  |  |  |  | | |  | |  | |  | |  | |  | |  | |  |
|  |  |  |  | | |  | |  | |  | |  | |  | |  | |  |
|  |  |  | |  | | |  | |  | |  | |  | |  | |  | |
|  |  |  | |  | | |  | |  | |  | |  | |  | |  | |

| **. Significance level p< 0,01 two tailed. | | | |  |  |  |  |  |  |  |
| --- | --- | --- | --- | --- | --- | --- | --- | --- | --- | --- |
| *. Significance level p< 0,05 two tailed. | | | |  |  |  |  |  |  |  |
| **Correlations AN-ow group** | | | | | | | | | | |
|  | | age | education | | bmi | BMI_P | Drive to Thinness | Bulimia | Body Dissatisfaction | Ineffectiveness |
| age | Pearson Correlation | 1 | ,974^**^ | | -,396 | -,662^**^ | -,014 | ,035 | -,223 | ,200 |
|  | Sign. two tailed |  | <,001 | | ,056 | <,001 | ,958 | ,893 | ,389 | ,441 |
|  | N | 24 | 24 | | 24 | 24 | 24 | 24 | 24 | 24 |
| education | Pearson Correlation | ,974^**^ | 1 | | -,320 | -,633^**^ | -,014 | ,035 | -,223 | ,200 |
|  | Sign. two tailed | <,001 |  | | ,128 | <,001 | ,958 | ,893 | ,389 | ,441 |
|  | N | 24 | 24 | | 24 | 24 | 24 | 24 | 24 | 24 |
| bmi | Pearson Correlation | -,396 | -,320 | | 1 | ,873^**^ | ,121 | -,092 | ,552^*^ | -,033 |
|  | Sign. two tailed | ,056 | ,128 | |  | <,001 | ,643 | ,727 | ,022 | ,900 |
|  | N | 24 | 24 | | 24 | 24 | 24 | 24 | 24 | 24 |
| BMI_P | Pearson Correlation | -,662^**^ | -,633^**^ | | ,873^**^ | 1 | ,190 | ,084 | ,525^*^ | -,117 |
|  | Sign. two tailed | <,001 | <,001 | | <,001 |  | ,465 | ,749 | ,030 | ,655 |
|  | N | 24 | 24 | | 24 | 24 | 24 | 24 | 24 | 24 |
| Drive to Thinness | Pearson Correlation | -,014 | -,014 | | ,121 | ,190 | 1 | ,384 | ,521^*^ | ,120 |
|  | Sign. two tailed | ,958 | ,958 | | ,643 | ,465 |  | ,128 | ,032 | ,645 |
|  | N | 24 | 24 | | 24 | 24 | 24 | 24 | 24 | 24 |
| Bulimia | Pearson Correlation | ,035 | ,035 | | -,092 | ,084 | ,384 | 1 | -,087 | ,067 |
|  | Sign. two tailed | ,893 | ,893 | | ,727 | ,749 | ,128 |  | ,739 | ,800 |
|  | N | 24 | 24 | | 24 | 24 | 24 | 24 | 24 | 24 |
| Body Dissatisfaction | Pearson Correlation | -,223 | -,223 | | ,552^*^ | ,525^*^ | ,521^*^ | -,087 | 1 | ,292 |
|  | Sign. two tailed | ,389 | ,389 | | ,022 | ,030 | ,032 | ,739 |  | ,256 |
|  | N | 24 | 24 | | 24 | 24 | 24 | 24 | 24 | 24 |
| Ineffectiveness | Pearson Correlation | ,200 | ,200 | | -,033 | -,117 | ,120 | ,067 | ,292 | 1 |
|  | Sign. two tailed | ,441 | ,441 | | ,900 | ,655 | ,645 | ,800 | ,256 |  |
|  | N | 24 | 24 | | 24 | 24 | 24 | 24 | 24 | 24 |
| Social Insecurity | Pearson Correlation | ,433 | ,433 | | -,032 | -,291 | ,125 | -,146 | ,261 | ,587^*^ |
|  | Sign. two tailed | ,083 | ,083 | | ,903 | ,257 | ,634 | ,576 | ,312 | ,013 |
|  | N | 24 | 24 | | 24 | 24 | 24 | 24 | 24 | 24 |
| Asceticism | Pearson Correlation | -,043 | -,043 | | -,134 | -,122 | ,063 | ,292 | ,223 | ,495^*^ |
|  | Sign. two tailed | ,870 | ,870 | | ,608 | ,640 | ,811 | ,255 | ,390 | ,043 |
|  | N | 24 | 24 | | 24 | 24 | 24 | 24 | 24 | 24 |
| Interosceptive Awareness | Pearson Correlation | -,042 | -,042 | | ,063 | ,152 | ,199 | ,472 | ,072 | ,535^*^ |
|  | Sign. two tailed | ,874 | ,874 | | ,810 | ,561 | ,444 | ,056 | ,785 | ,027 |
|  | N | 24 | 24 | | 24 | 24 | 24 | 24 | 24 | 24 |
| Interpersonal Distrust | Pearson Correlation | ,550^*^ | ,550^*^ | | -,325 | -,618^**^ | -,289 | -,153 | -,350 | ,410 |
|  | Sign. two tailed | ,022 | ,022 | | ,203 | ,008 | ,260 | ,557 | ,168 | ,102 |
|  | N | 24 | 24 | | 24 | 24 | 24 | 24 | 24 | 24 |
| Difficulty in Identifying Feelings | Pearson Correlation | ,014 | ,014 | | ,286 | ,280 | ,205 | ,288 | ,448 | -,034 |
|  | Sign. two tailed | ,960 | ,960 | | ,283 | ,293 | ,447 | ,280 | ,082 | ,899 |
|  | N | 24 | 24 | | 24 | 24 | 24 | 24 | 24 | 24 |
| PATERNAL CARE | Pearson Correlation | ,384 | ,384 | | -,481 | -,529^*^ | -,148 | -,291 | -,425 | -,234 |
|  | Sign. two tailed | ,128 | ,128 | | ,051 | ,029 | ,572 | ,257 | ,089 | ,366 |
|  | N | 24 | 24 | | 24 | 24 | 24 | 24 | 24 | 24 |
| Total Depression | Pearson Correlation | -,006 | -,006 | | ,276 | ,228 | ,340 | ,432 | ,481 | ,446 |
|  | Sign. two tailed | ,983 | ,983 | | ,283 | ,378 | ,182 | ,084 | ,050 | ,073 |
|  | N | 24 | 24 | | 24 | 24 | 24 | 24 | 24 | 24 |
| Cognitive Depression | Pearson Correlation | ,065 | ,065 | | ,253 | ,171 | ,342 | ,354 | ,513^*^ | ,418 |
|  | Sign. two tailed | ,805 | ,805 | | ,326 | ,513 | ,179 | ,163 | ,035 | ,095 |
|  | N | 24 | 24 | | 24 | 24 | 24 | 24 | 24 | 24 |
| Somatic Depression | Pearson Correlation | -,081 | -,081 | | ,278 | ,272 | ,311 | ,480 | ,409 | ,440 |
|  | Sign. two tailed | ,758 | ,758 | | ,280 | ,291 | ,225 | ,051 | ,103 | ,077 |
|  | N | 24 | 24 | | 24 | 24 | 24 | 24 | 24 | 24 |

| **Correlations** | | | | | | | | | |
| --- | --- | --- | --- | --- | --- | --- | --- | --- | --- |
|  | | Social Insecurity | Asceticism | Interosceptive Awareness | Interpersonal Distrust | Difficulty in Identifying Feelings | PATERNAL CARE | Total Depression | Cognitive Depression |
| age | Pearson Correlation | ,433 | -,043 | -,042 | ,550^*^ | ,014 | ,384 | -,006 | ,065 |
|  | Sign. two tailed | ,083 | ,870 | ,874 | ,022 | ,960 | ,128 | ,983 | ,805 |
|  | N | 24 | 24 | 24 | 24 | 24 | 24 | 24 | 24 |
| education | Pearson Correlation | ,433 | -,043 | -,042 | ,550^*^ | ,014 | ,384 | -,006 | ,065 |
|  | Sign. two tailed | ,083 | ,870 | ,874 | ,022 | ,960 | ,128 | ,983 | ,805 |
|  | N | 24 | 24 | 24 | 24 | 24 | 24 | 24 | 24 |
| bmi | Pearson Correlation | -,032 | -,134 | ,063 | -,325 | ,286 | -,481 | ,276 | ,253 |
|  | Sign. two tailed | ,903 | ,608 | ,810 | ,203 | ,283 | ,051 | ,283 | ,326 |
|  | N | 24 | 24 | 24 | 24 | 24 | 24 | 24 | 24 |
| BMI_P | Pearson Correlation | -,291 | -,122 | ,152 | -,618^**^ | ,280 | -,529^*^ | ,228 | ,171 |
|  | Sign. two tailed | ,257 | ,640 | ,561 | ,008 | ,293 | ,029 | ,378 | ,513 |
|  | N | 24 | 24 | 24 | 24 | 24 | 24 | 24 | 24 |
| Drive to Thinness | Pearson Correlation | ,125 | ,063 | ,199 | -,289 | ,205 | -,148 | ,340 | ,342 |
|  | Sign. two tailed | ,634 | ,811 | ,444 | ,260 | ,447 | ,572 | ,182 | ,179 |
|  | N | 24 | 24 | 24 | 24 | 24 | 24 | 24 | 24 |
| Bulimia | Pearson Correlation | -,146 | ,292 | ,472 | -,153 | ,288 | -,291 | ,432 | ,354 |
|  | Sign. two tailed | ,576 | ,255 | ,056 | ,557 | ,280 | ,257 | ,084 | ,163 |
|  | N | 24 | 24 | 24 | 24 | 24 | 24 | 24 | 24 |
| Body Dissatisfaction | Pearson Correlation | ,261 | ,223 | ,072 | -,350 | ,448 | -,425 | ,481 | ,513^*^ |
|  | Sign. two tailed | ,312 | ,390 | ,785 | ,168 | ,082 | ,089 | ,050 | ,035 |
|  | N | 24 | 24 | 24 | 24 | 24 | 24 | 24 | 24 |
| Ineffectiveness | Pearson Correlation | ,587^*^ | ,495^*^ | ,535^*^ | ,410 | -,034 | -,234 | ,446 | ,418 |
|  | Sign. two tailed | ,013 | ,043 | ,027 | ,102 | ,899 | ,366 | ,073 | ,095 |
|  | N | 24 | 24 | 24 | 24 | 24 | 24 | 24 | 24 |
| Social Insecurity | Pearson Correlation | 1 | ,272 | ,027 | ,639^**^ | -,218 | -,092 | ,385 | ,462 |
|  | Sign. two tailed |  | ,290 | ,917 | ,006 | ,417 | ,726 | ,127 | ,062 |
|  | N | 24 | 24 | 24 | 24 | 24 | 24 | 24 | 24 |
| Asceticism | Pearson Correlation | ,272 | 1 | ,306 | ,292 | ,203 | -,153 | ,453 | ,412 |
|  | Sign. two tailed | ,290 |  | ,232 | ,256 | ,451 | ,557 | ,068 | ,100 |
|  | N | 24 | 24 | 24 | 24 | 24 | 24 | 24 | 24 |
| Interosceptive Awareness | Pearson Correlation | ,027 | ,306 | 1 | -,009 | ,414 | -,232 | ,341 | ,205 |
|  | Sign. two tailed | ,917 | ,232 |  | ,971 | ,111 | ,370 | ,180 | ,429 |
|  | N | 24 | 24 | 24 | 24 | 24 | 24 | 24 | 24 |
| Interpersonal Distrust | Pearson Correlation | ,639^**^ | ,292 | -,009 | 1 | -,544^*^ | ,229 | -,164 | -,126 |
|  | Sign. two tailed | ,006 | ,256 | ,971 |  | ,029 | ,376 | ,530 | ,629 |
|  | N | 24 | 24 | 24 | 24 | 24 | 24 | 24 | 24 |
| Difficulty in Identifying Feelings | Pearson Correlation | -,218 | ,203 | ,414 | -,544^*^ | 1 | -,030 | ,496 | ,441 |
|  | Sign. two tailed | ,417 | ,451 | ,111 | ,029 |  | ,911 | ,051 | ,088 |
|  | N | 24 | 24 | 24 | 24 | 24 | 24 | 24 | 24 |
| PATERNAL CARE | Pearson Correlation | -,092 | -,153 | -,232 | ,229 | -,030 | 1 | -,512^*^ | -,393 |
|  | Sign. two tailed | ,726 | ,557 | ,370 | ,376 | ,911 |  | ,036 | ,118 |
|  | N | 24 | 24 | 24 | 24 | 24 | 24 | 24 | 24 |
| Total Depression | Pearson Correlation | ,385 | ,453 | ,341 | -,164 | ,496 | -,512^*^ | 1 | ,964^**^ |
|  | Sign. two tailed | ,127 | ,068 | ,180 | ,530 | ,051 | ,036 |  | <,001 |
|  | N | 24 | 24 | 24 | 24 | 24 | 24 | 24 | 24 |
| Cognitive Depression | Pearson Correlation | ,462 | ,412 | ,205 | -,126 | ,441 | -,393 | ,964^**^ | 1 |
|  | Sign. two tailed | ,062 | ,100 | ,429 | ,629 | ,088 | ,118 | <,001 |  |
|  | N | 24 | 24 | 24 | 24 | 24 | 24 | 24 | 24 |
| Somatic Depression | Pearson Correlation | ,271 | ,461 | ,459 | -,190 | ,516^*^ | -,598^*^ | ,958^**^ | ,847^**^ |
|  | Sign. two tailed | ,293 | ,062 | ,064 | ,464 | ,041 | ,011 | <,001 | <,001 |
|  | N | 24 | 24 | 24 | 24 | 24 | 24 | 24 | 24 |

| **Correlations** | | | |  |  |  |  |  |  |  |
| --- | --- | --- | --- | --- | --- | --- | --- | --- | --- | --- |
|  | | Somatic Depression | |  |  |  |  |  |  |  |
| age | Pearson Correlation | -,081 | |  |  |  |  |  |  |  |
|  | Sign. two tailed | ,758 | |  |  |  |  |  |  |  |
|  | N | 24 | |  |  |  |  |  |  |  |
| education | Pearson Correlation | -,081 | |  |  |  |  |  |  |  |
|  | Sign. two tailed | ,758 | |  |  |  |  |  |  |  |
|  | N | 24 | |  |  |  |  |  |  |  |
| bmi | Pearson Correlation | ,278 | |  |  |  |  |  |  |  |
|  | Sign. two tailed | ,280 | |  |  |  |  |  |  |  |
|  | N | 24 | |  |  |  |  |  |  |  |
| BMI_P | Pearson Correlation | ,272 | |  |  |  |  |  |  |  |
|  | Sign. two tailed | ,291 | |  |  |  |  |  |  |  |
|  | N | 24 | |  |  |  |  |  |  |  |
| Drive to Thinness | Pearson Correlation | ,311 | |  |  |  |  |  |  |  |
|  | Sign. two tailed | ,225 | |  |  |  |  |  |  |  |
|  | N | 24 | |  |  |  |  |  |  |  |
| Bulimia | Pearson Correlation | ,480 | |  |  |  |  |  |  |  |
|  | Sign. two tailed | ,051 | |  |  |  |  |  |  |  |
|  | N | 24 | |  |  |  |  |  |  |  |
| Body Dissatisfaction | Pearson Correlation | ,409 | |  |  |  |  |  |  |  |
|  | Sign. two tailed | ,103 | |  |  |  |  |  |  |  |
|  | N | 24 | |  |  |  |  |  |  |  |
| Ineffectiveness | Pearson Correlation | ,440 | |  |  |  |  |  |  |  |
|  | Sign. two tailed | ,077 | |  |  |  |  |  |  |  |
|  | N | 24 | |  |  |  |  |  |  |  |
| Social Insecurity | Pearson Correlation | ,271 | |  |  |  |  |  |  |  |
|  | Sign. two tailed | ,293 | |  |  |  |  |  |  |  |
|  | N | 24 | |  |  |  |  |  |  |  |
| Asceticism | Pearson Correlation | ,461 | |  |  |  |  |  |  |  |
|  | Sign. two tailed | ,062 | |  |  |  |  |  |  |  |
|  | N | 24 | |  |  |  |  |  |  |  |
| Interosceptive Awareness | Pearson Correlation | ,459 | |  |  |  |  |  |  |  |
|  | Sign. two tailed | ,064 | |  |  |  |  |  |  |  |
|  | N | 24 | |  |  |  |  |  |  |  |
| Interpersonal Distrust | Pearson Correlation | -,190 | |  |  |  |  |  |  |  |
|  | Sign. two tailed | ,464 | |  |  |  |  |  |  |  |
|  | N | 24 | |  |  |  |  |  |  |  |
| Difficulty in Identifying Feelings | Pearson Correlation | ,516^*^ | |  |  |  |  |  |  |  |
|  | Sign. two tailed | ,041 | |  |  |  |  |  |  |  |
|  | N | 24 | |  |  |  |  |  |  |  |
| PATERNAL CARE | Pearson Correlation | -,598^*^ | |  |  |  |  |  |  |  |
|  | Sign. two tailed | ,011 | |  |  |  |  |  |  |  |
|  | N | 24 | |  |  |  |  |  |  |  |
| Total Depression | Pearson Correlation | ,958^**^ | |  |  |  |  |  |  |  |
|  | Sign. two tailed | <,001 | |  |  |  |  |  |  |  |
|  | N | 24 | |  |  |  |  |  |  |  |
| Cognitive Depression | Pearson Correlation | ,847^**^ | |  |  |  |  |  |  |  |
|  | Sign. two tailed | <,001 | |  |  |  |  |  |  |  |
|  | N | 24 | |  |  |  |  |  |  |  |
| Somatic Depression | Pearson Correlation | 1 | |  |  |  |  |  |  |  |
|  | Sign. two tailed |  | |  |  |  |  |  |  |  |
|  | N | 24 | |  |  |  |  |  |  |  |
|  |  |  |  | |  |  |  |  |  |  |
|  |  |  |  | |  |  |  |  |  |  |

|  |  |  |  |  |  |  |  |  |  |
| --- | --- | --- | --- | --- | --- | --- | --- | --- | --- |
|  |  |  |  |  |  |  |  |  |  |

| **. Significance level p< 0,01 two tailed. |
| --- |
| *. Significance level p< 0,05 two tailed. |
